# MutationAligner: a resource of recurrent mutation hotspots in protein domains in cancer

**DOI:** 10.1093/nar/gkv1132

**Published:** 2015-11-20

**Authors:** Nicholas Paul Gauthier, Ed Reznik, Jianjiong Gao, Selcuk Onur Sumer, Nikolaus Schultz, Chris Sander, Martin L. Miller

**Affiliations:** 1 Computational Biology Center, Memorial Sloan Kettering Cancer Center, New York, NY 10065, USA; 2 Cancer Research UK, Cambridge Institute, University of Cambridge, Cambridge, CB2 0RE, UK

## Abstract

The MutationAligner web resource, available at http://www.mutationaligner.org, enables discovery and exploration of somatic mutation hotspots identified in protein domains in currently (mid-2015) more than 5000 cancer patient samples across 22 different tumor types. Using multiple sequence alignments of protein domains in the human genome, we extend the principle of recurrence analysis by aggregating mutations in homologous positions across sets of paralogous genes. Protein domain analysis enhances the statistical power to detect cancer-relevant mutations and links mutations to the specific biological functions encoded in domains. We illustrate how the MutationAligner database and interactive web tool can be used to explore, visualize and analyze mutation hotspots in protein domains across genes and tumor types. We believe that MutationAligner will be an important resource for the cancer research community by providing detailed clues for the functional importance of particular mutations, as well as for the design of functional genomics experiments and for decision support in precision medicine. MutationAligner is slated to be periodically updated to incorporate additional analyses and new data from cancer genomics projects.

## INTRODUCTION

Through major tumor profiling projects, such as the The Cancer Genome Atlas (TCGA), it is becoming clear that the mutational landscape in cancer is extraordinarily complex (1). Recent analysis of mutations across cancer types (‘pan-cancer’ analysis) has revealed that relatively few genes are recurrently mutated in a high proportion of samples (2) above expectation from a random distribution without clonal selection. These genes are hypothetical or known cancer genes. At the other end of the spectrum there exists a long tail of infrequently mutated genes (3). For such rare mutations, recurrence analysis is insufficient to distinguish functional mutations from background passenger alterations. In some cases prior independent knowledge of the oncogenic potential of rare mutations is available, but in many cases such knowledge is borderline or not available. Enhancing the computational-analytic methods for establishing the identity of infrequent mutations that play causative roles in cancer is not only an important scientific question for understanding tumorigenesis, but is also essential to the development of personalized cancer therapies.

A clue to understanding the potential consequence of infrequent mutations may come from analyzing genetic alterations across genes involved in similar biological processes. One powerful method for systematically assessing the common biological functions of genes is through the analysis of protein domains. Protein domains are evolutionarily conserved and structurally related functional regions encoded in the primary sequence of proteins (4,5). Proteins with shared domains (protein families) have a high degree of sequence similarity, which is often detected and analyzed using multiple sequence analysis and hidden Markov models (HMMs). Large repositories of information about protein domains include the manually curated database for protein families (Pfam-A), which contains sequence information as well as annotations about the known biological roles and functions of domains in various species (6).

To address the critical challenge of associating function with rare mutations in cancer, we present a comprehensive web-based resource of the analysis of mutations in protein domains in cancer. This resource, called ‘MutationAligner’, is associated with our recent work describing a new analytical framework that uses domains, rather than individual genes, as the basis for the discovery of functionally relevant mutations in cancer (7). MutationAligner enhances statistical power by aggregating mutations across sets of genes sharing a common domain. In doing so, the website also enables novel hypothesis generation by associating rare mutations of unknown function with well-characterized mutations in homologous domain positions.

In previous work, we and others have utilized the sequence similarity encoded in domains to discover ‘domain hotspots’, which can be defined as recurrent mutations affecting conserved residues in 3D structures or conserved positions in multiple sequence alignments of protein domains (7–9). In the MutationAligner approach (7), we systematically analyzed nearly 500 000 missense mutations from 22 TCGA-profiled tumor types deposited in the cBioPortal (10) by tallying mutations across sequence alignments of protein domains. We implemented a binomial statistical test to identify significant domain hotspots. Yue *et al*. developed the ‘mCluster’ approach (http://research-pub.gene.com/mcluster) in which around 9000 somatic cancer mutations, 19 000 germline disease mutations and 17 000 other annotations of amino acid residues were analyzed in the context of sequence alignments of domains (9). mCluster uses similar binomial statistics to identify domain hotspots or ‘clusters’, except mCluster performs the cluster detection using a step-down approach analyzing the position with the largest number of mutations first followed by the next largest and so forth. In the ‘DMDM’ (Domain mapping of disease mutations) approach, Peterson *et al*. analyzed around 100 000 polymorphisms and disease mutations in domains (8). The data are presented at http://bioinf.umbc.edu/dmdm/ using sequence logo plots of aligned domains and associated histograms of mutation counts, although no statistics are provided. Due in part to the scarcity of data available at the time of analysis, the mCluster and DMDM methods did not include a systematic pan-cancer analysis of mutation hotspots in domains. Other bioinformatics analyses of mutations in domains performed more recently (11–13) have focused on characterizing and identifying mutations enriched across the full length of domains and do therefore not include multiple sequence analysis and hotspot detection across domain-containing genes.

In this work, we (i) describe the MutationAligner database and how other resources are combined and integrated (Pfam-A, cBioPortal and TCGA) (1,6,10), (ii) provide technical details about the database construction and software architecture and (iii) provide guidelines and illustrate with an example of how exploring the database can reveal biological insights of mutations in domains in cancer. The web resource is linked to the cBioPortal for cancer genomics data and is freely available at http://www.mutationaligner.org. We believe that MutationAligner will be a valuable resource to computational, experimental and clinical cancer researchers.

## DATABASE CONSTRUCTION AND SOFTWARE ARCHITECTURE

### Data collection and analysis

**Figure 1.**
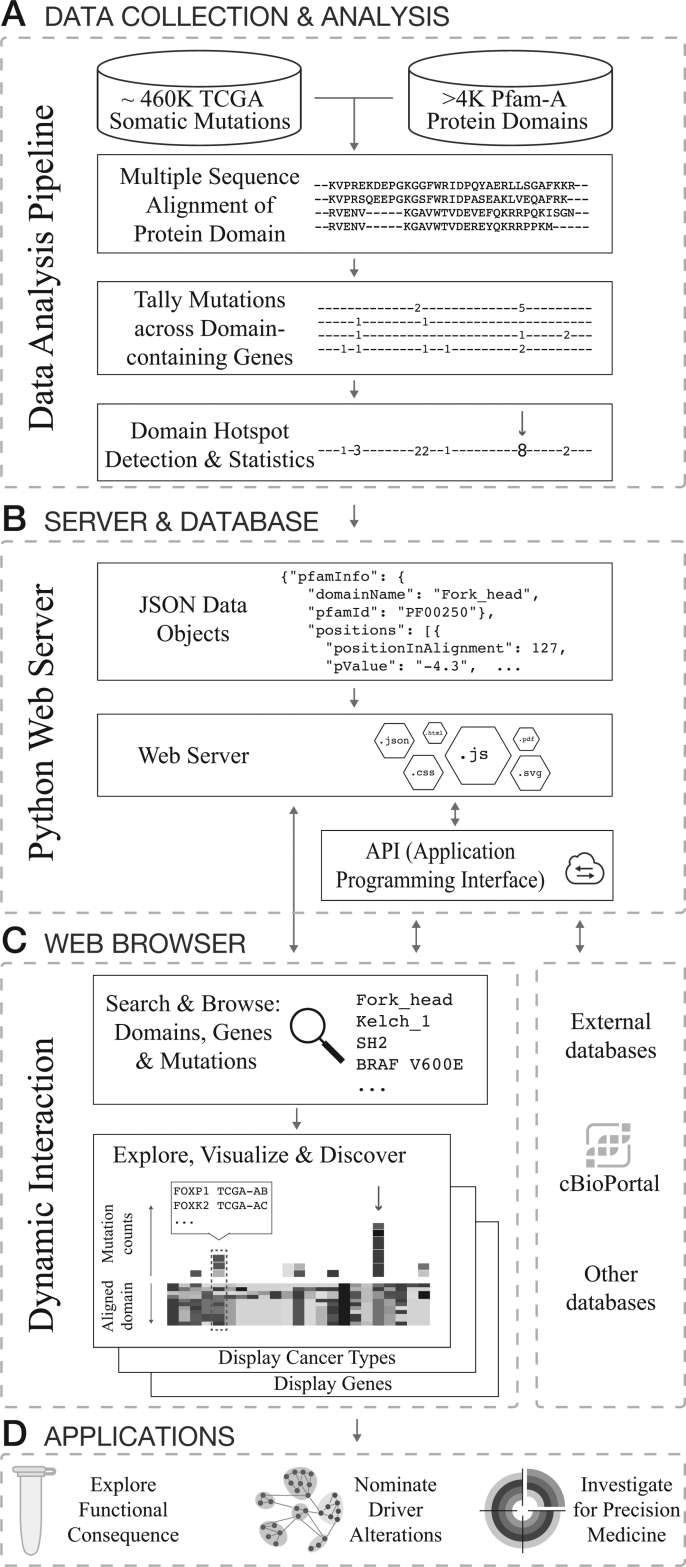
Database construction and software architecture of mutationaligner.org. (**A**) Integrated analysis of cancer mutations from TCGA and protein domains from Pfam-A. (**B**) Identified domain mutation hotspots and analysis results are stored and served from a python webserver via a documented Application Programming Interface (API, see main text). The webserver also provides the files required for the single page javascript application. (**C**) Analysis results are provided interactively in the user's web browser (client-side) through a rich, single page javascript application. (**D**) Examples of potential follow-on applications based on observations made using MutationAligner.

The data and analysis pipeline behind the MutationAligner database is illustrated in Figure 1A and is described in detail elsewhere (7). Briefly, around 460 000 missense mutations from 5496 exome-sequenced tumor-normal pairs of 22 different tumor types studied by the TGCA consortium were obtained from the cBioPortal (10) in the format of TCGA level 3 variant data. Missense mutations were mapped to protein sequences and protein domains annotated by Pfam-A version 26 (6). Pfam-A domains were excluded from the analysis if (i) no missense mutations were present, (ii) the Pfam-A expectancy score (*e*-value) was greater than 1e^−5^ or (iii) the domain was only present as one instance in the human genome. In total, 4401 Pfam-A domains were analyzed by multiple sequence alignment of domain regions across protein families using the MathWorks multialign package with BLOSUM80 as scoring matrix and default parameters. As sequences of domains in paralogous genes within the same species (human) are closely related, the BLOSUM80 matrix was chosen as it is designed for comparing less divergent sequences than other matrices (e.g. BLOSUM45). Mutations were tallied across samples and across domain-containing genes using the coordinates of the multiple sequence alignment. To identify putative hotspots for mutations within a domain, we used a binomial test, taking into account the length and total number of mutations observed in the domain. This test generated a *P*-value by comparing the number of mutations observed at that domain position to what would be observed by chance assuming a random distribution of mutations. In total, 17 273 positions in the domain sequence alignments (‘positions’) were analyzed based on the following criteria: (i) at least two missense mutations occurred at each position and (ii) at least three quarters of the domain alignments were non-gaps at each position. All *P*-values were adjusted for multiple hypothesis testing using the stringent Bonferroni correction method. We identified 82 significant (adjusted *P* < 0.05) hotspots in 42 different domains (7).

### Webserver and application programming interface (API)

The primary function of the MutationAligner webserver is to deliver a single HTML, javascript and cascading style sheet (Cascading Style Sheet) file to client web browsers (see Interactive Client-Side below). In addition, the website provides data access to other software systems through a representational state transfer (REST) application programming interface (API). This REST API returns Javascript Object Notation (JSON) files and currently supports the following three methods:

1. http://mutationaligner.org/api/positions
  Returns a single object with details of all analyzed positions.
2. http://mutationaligner.org/api/domains
  Returns an array containing a list of all domains analyzed that contain an interactive domain details page.
3. http://mutationaligner.org/api/domains/**domainname**
  Returns a single object for the **domainname** entered or Not Found error (HTTP 404) if the domain does not exist or was not analyzed. Both Pfam accession codes and domain names are supported.

Documentation about each JSON format is available at http://mutationaligner.org/API.

### Interactive user interface

All data interaction and visualization are driven by a rich Single Page Application (SPA) powered by the AngularJS javascript framework. The website provides several views for browsing and interacting with the analysis results and the raw mutation data. Upon visiting mutationaligner.org, users are presented with a browsable table that contains information and key analysis results for all analyzed positions. This table is populated from the positions JSON file (see API#1 above). Typing into the search box dynamically updates the table to display positions that contain mutations in particular genes, cancer types or domains of interest. Clicking on the domain name or chart icon of any position loads the domain details page. This page is populated from the requested domain's JSON file (see API#3 above), and allows users to display and interact with mutational profiles of individual domains. Mutation histograms are produced in Scalable Vector Graphics (SVG) format with the D3 javascript library, and the multiple sequence alignments are drawn with custom canvas drawing code. The website has been tested with the latest version of all major browsers (Firefox, Google Chrome, Internet Explorer and Safari).

## EXPLORING MUTATION HOTSPOTS IN DOMAINS

MutationAligner can be used interactively as a resource to explore and analyze the incidence of mutations across functionally related genes. In order to highlight some of the features of the website, we describe a typical use case scenario of a researcher interested in a particular gene (the Kelch_1 domain-containing gene *KEAP1*) and its role in cancer.

The *KEAP1* gene regulates the transcription factor *NRF2* by acting as a substrate adaptor protein for the Cul3-RBX1 E3-ubiquitin ligase complex (14). *KEAP1* is cysteine-rich, and modification of cysteine residues due to oxidative stress can induce a conformational change in *KEAP1*, leading to the nuclear translocation of *NRF2* and up-regulation of anti-oxidant stress response genes (15). *KEAP1* is composed of a series of six repeated Kelch_1 domains, and the mutation of residues in this domain have been shown to lead to dysregulated activation of *NRF2* (14).

The Kelch_1 domain encodes a Kelch motif, typically composed of ≈40–50 amino acids, which form a four-stranded β-sheet. Kelch motifs are typically arranged in a series of tandem repeats, which then organize into a β-propeller tertiary structure often mediating protein-protein interactions (16).

### Search & explore

**Figure 2.**
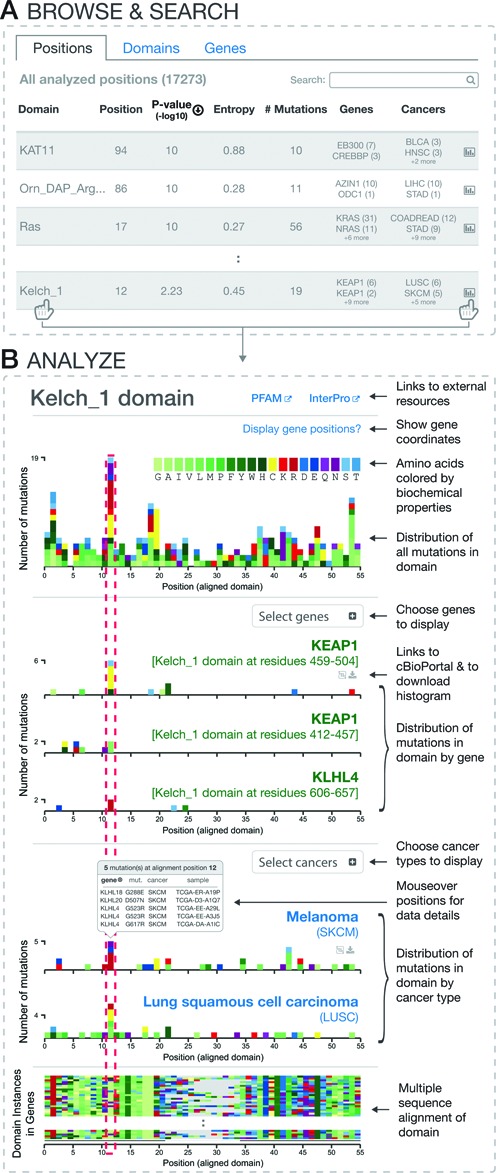
Interactive exploration of mutation hotspots in protein domains. (**A**) Searchable and sortable tables of all domain mutation hotspots by positions, domains and genes. (**B**) Domain details page with multiple sequence alignment and interactive mutation histograms.

On the mutationaligner.org homepage, users can query a searchable and sortable table with all mutations analyzed in the context of protein domains, providing an effective way of identifying domain mutations in specific genes or tumor types of interest (Figure 2A).

The table of all analyzed positions (positions tab) is dynamically updated to show matching search results of the >17K domain mutations analyzed and provides the user with information about (i) the specific position in the multiple sequence alignment that is mutated; (ii) the hotspot significance level (–log10 of the *P*-value); (iii) the entropy score that estimates how uniformly mutations are spread across domain-containing genes, where a high score indicates that multiple genes are mutated in the particular position (the entropy score is normalized to the domain family size, with a maximum score of 1 signifying that mutation counts are the same for all genes); (iv) the number of identified mutations; (v) information on which genes are mutated; (vi) information on which cancer types are mutated (7).

In addition to the positions tab, the domains tab and genes tab provide users with an option to browse and search for particular domains and genes. The statistics provided for these tabs are similar to those described above, except only the position with the best *P*-value or highest entropy score (adjustable via a toggle button) is displayed. Of note, not all domains and genes in the human genome are listed as some domains, genes and mutations were filtered out using the exclusion criteria stated in Data Collection and Analysis section. For example, canonical hotspots in well-known cancer-associated genes, such as *APC*, may not be included as either the mutation type was not analyzed (e.g. truncating mutations were excluded) and/or there were no missense mutation hotspots in Pfam-A annotated domains within the gene.

To facilitate browsing and exploration, all analyzed data can be ranked by clicking and sorting the columns to inspect particular mutations of interest. In the Kelch_1 domain, for example, position 12 is found as the most significant hotspot with mutations in multiple genes including *KEAP1*. That mutations in position 12 are contributed by multiple genes is also indicated by the relatively high entropy score (∼0.5), which may indicate that this particular hotspot is difficult to detect using a gene-based approach but only revealed in the context of a domain analysis.

To explore the mutations in Kelch_1 further, users can click on the domain name or chart icon (Figure 2A, bottom), bringing the user to the domain details page.

### Visualize & analyze

The details page of user-selected domains visualizes mutations in the context of domain sequence alignments and allows users to analyze mutations across selected genes and tumor types.

Figure 2B depicts a typical domain details page summarizing information for mutations in the selected Kelch_1 domain. The header of the details page lists the name of the domain and contains relevant information such as the domain description and links to Pfam and InterPro (17). A summary histogram at the top of the page depicts the distribution of aligned mutations, summed across all cancer types over all genes containing the Kelch_1 domain (occurs 116 times in 43 genes). The color of each unit box in the histogram indicates the identity of the new amino acid that is generated by the missense mutation. Amino acids are grouped and color coded by their biochemical properties, enabling user to identify general patterns of mutational changes. For example, in the tallest peak (position 12), a large proportion of the new amino acids generated by mutations are positively charged, basic amino acids (R and K, red) and cysteines (C, yellow). All histograms are interactive, displaying summary data tables upon mouseover and highlighting particular positions across all currently displayed charts in response to user clicks. This highlight makes it easy to compare aligned positions in different genes and cancer types. To display gene-specific sequence coordinates when hovering over the domain positions in the mutation histograms, the user can select genes of interest via the ‘display gene positions’ option.

For illustrative purposes, we have selected the most prominant peak in the Kelch_1 domain, which occurs at position 12 of the multiple sequence alignment (red dotted line in Figure 2B). The peak at position 12 is an aggregate of homologous mutations of the Kelch_1 domain across multiple genes. Among eight genes that have mutations in position 12, *KEAP1* has the most mutations, which can be displayed in more detail using the ‘Select gene’ option (note that only five of the six Kelch_1 domains in *KEAP1* were analyzed as the first instance was excluded based on high *e*-value). Displaying *KEAP1*'s fourth Kelch_1 domain, which spans amino acid positions 459–504, shows six mutations in residue R470 with four being R470C mutations. The *KEAP1* R470C mutation has been reported as a ‘super-binder’ in the literature, leading to enhanced *KEAP1* and *NRF2* interaction and hypomorphic regulation of *NRF2* by *KEAP1* (18). Interestingly, two mutations occur in *KEAP1* at position G423 in the third Kelch_1 repeat (residues 412–457), and these align precisely with the R470 mutation hotspot. As these two repeats are mutated at analogous positions and multiple repeats are needed to form the functional Kelch_1 β-propeller, these observations indicate that G423 mutations are likely functionally relevant despite being rare mutation events. Supporting this, Hast *et al*. have reported that the non-canonical G423 mutation in *KEAP1* has an identical ‘super-binder’ effect as the R470C mutation (18).

By using the ‘Select cancers’ option, users can view the distribution of mutations for individual tumor types. For Kelch_1 this view shows that lung cancers in general have many mutations distributed over the domain. For position 12 specifically, lung squamous cell carcinoma (LUSC) has six mutations (LUSC, Figure 2B) while lung adenocarcinoma (LUAD) has three with the majority of these position 12 mutations being in *KEAP1*. Accordingly, *KEAP1* has previously been reported as significantly altered in large-scale sequencing projects in lung adenocarcinoma (19) and lung squamous cell carcinoma (20).

### Discover

The MutationAligner tool offers the possibility to draw new and unexpected links between observations of mutations across different genes as mutations may often affect equivalent residues of domains of paralogous genes. Analyzing mutations across different user-selected tumor types also offers the ability to transfer knowledge from one context to another, particularly when recurrent mutations in one cancer type overlaps with more infrequent mutations in other cancer types.

For example, in the case of Kelch_1, while most mutations at position 12 were in lung cancer, using MutationAligner it is clear that melanomas (SKCM) also contain mutations in the same position (five mutations, Figure 2B). To examine the details of individual mutations in SKCM, one can mouseover the position and see the mutated genes, corresponding residues and samples. Interestingly, out of the five mutations found in melanoma in position 12, none of the mutations occur in *KEAP1*, but rather these mutations are spread across *KLHL4* (three mutations), *KLHL18* and *KLHL20* (see box in Figure 2B). These KLHL genes were not highlighted in a recent cancer genomics marker paper on melanoma by the TCGA consortium (21).

The color coded multiple sequence alignment on the bottom of the domain details page reveals that the Kelch_1 domain has a high degree of sequence similarity across paralogous genes in the human genome. Thus, based on the structure-function relationship encoded Kelch_1, this indicates that the rare mutations in melanoma (e.g. *KLHL4* G523R) could alter Kelch_1-mediated function as they affect equivalent positions to the canonical *KEAP1* hotspot mutations reported in lung cancer. Taken together, the observation that mutations in the Kelch_1 domain of *KLHL4* align precisely with hotspot mutations in *KEAP1*, in tandem with a lack of reports of oncogenic mutations in *KLHL4* in melanoma, make these mutations an appealing subject for future investigation.

## DISCUSSION

The end-product of tumor sequencing pipelines is a list of mutations of putatively unknown consequence. Distilling meaning from this list, by annotating variants with their likely contribution to the malignancy, is critical to understanding cancer biology and designing therapeutic protocols. While the TCGA has succeeded in identifying many recurrent alterations in tumors, a number of studies have highlighted the ‘long tail’ phenomenon in cancer where many genes are infrequently mutated, making it difficult to distinguish between non-functional passenger mutations and true oncogenic driver mutations (2,3,22).

In this work, we present MutationAligner, a rich web resource that allows researchers to search, browse and analyze cancer mutations in the context of protein domains. By taking a domain-centric approach, we identify new mutation hotspots in domains of genes not previously associated with cancer and predict the functional role of many rare mutations (7). With the current data set of 22 tumor types and 5496 samples (mid*-*2015) we have identified 82 significant domain hotspots. This number is likely to increase as we further develop the analysis methodology and as more tumor types and samples become available.

As an example of the utility of the resource, we show how canonical hotspot mutations in *KEAP1* in lung cancer align with rare, and putatively functional, mutations in other Kelch_1-containing genes in melanoma. We believe that users will discover other relevant domain mutations, providing interesting hypotheses for further experimental followup, for example investigating the effect of mutations at the signaling pathway level and understanding how such knowledge can be applied clinically (Figure 1D). As more cancers are sequenced this framework will help point to new alterations in domains with potential functional impact.

We plan several future directions for MutationAligner. First, we plan to add several features such as displaying the domain structure for individual genes. Second, we plan to strengthen the connection with the cBioPortal for cancer genomics data. Although bidirectional hyperlinks have already been established, we would like to integrate more context dependent linking. For example, we would like to provide a mechanism by which cBioPortal can embed (for particular genes or cancer types) mutation histograms and domain sequence alignments generated by MutationAligner. Finally, we will continuously update the website with new analyses and cancer genomics data as they become available.
